# BRIDGING POSTCOLONIAL STUDIES AND AGING STUDIES THROUGH INTERSECTIONAL THEORY

**DOI:** 10.1093/geroni/igae098.0876

**Published:** 2024-12-31

**Authors:** Eva-Maria Trinkaus, Ulla Kriebernegg, Ulla Kriebernegg

**Affiliations:** Chair; Co-Chair; Discussant

## Abstract

Kimberlé Crenshaw (1991), who coined the term ‘intersectionality,’ described the need for a different perspective and emphasized the need for new critical thinking in order to address structural inequality. Adding to her matrix of intersecting inequalities of race, class, and gender, Calasanti and Slevin (2001) argue that “[a]geism interacts with other types of discrimination – sexism, racism, classism, and heterosexim – in ways that make the experience of ageism both similar and different across various groups,” bringing ‘age’ to the table. Taking into consideration that the fields of Aging Studies and Postcolonial Studies both address social and structural inequalities in the context of intersectionality, this symposium critically investigates the ways in which these fields of research intersect and make theoretical approaches fruitful for discussion by bringing Cultural Age Studies, Literary Gerontology, and Medical Ethics into conversation.

